# Temporal Viability of *Aedes aegypti* and *Aedes albopictus* Eggs Using Two Hygroscopic Substances as Preservatives under a Sterile Insect Technique (SIT) Program in Southern Mexico

**DOI:** 10.3390/insects13010015

**Published:** 2021-12-22

**Authors:** Eunice Nayeli Martínez-García, Esteban E. Díaz-González, Carlos F. Marina, J. Guillermo Bond, Jorge J. Rodríguez-Rojas, Gustavo Ponce-García, Rosa M. Sánchez-Casas, Ildefonso Fernández-Salas

**Affiliations:** 1 Facultad de Ciencias Biológicas, Universidad Autónoma de Nuevo León (UANL), Av. Universidad s/n, Cd. Universitaria, San Nicolas de los Garza 66455, Nuevo Leon, Mexico; naye_323@hotmail.com (E.N.M.-G.); gustavo.poncegc@uanl.edu.mx (G.P.-G.); 2 Servicios de Salud de Nuevo León, Laboratorio Estatal de Salud Pública, Serafín Peña 2211, Guadalupe 67180, Nuevo Leon, Mexico; esteban.diaz@saludnl.gob.mx; 3 Centro Regional de Investigación en Salud Pública (CRISP), Instituto Nacional de Salud Pública (INSP), 4a Av. Norte esquina 19 Calle Poniente s/n, Tapachula 30700, Chiapas, Mexico; fmarina@insp.mx (C.F.M.); gbond@insp.mx (J.G.B.); 4 Unidad de Patógenos y Vectores, Centro de Investigación y Desarrollo en Ciencias de la Salud (CIDICS), Universidad Autónoma de Nuevo León, Dr. Carlos Canseco s/n esquina Dr. J. E. González, Mitras Centro, Monterrey 64460, Nuevo Leon, Mexico; jorge.rodriguezr@uanl.mx (J.J.R.-R.); rosa.sanchezcss@uanl.edu.mx (R.M.S.-C.); 5 Facultad de Medicina Veterinaria y Zootecnia, Universidad Autónoma de Nuevo León, General Francisco Villa 20, Hacienda del Cañada, General Escobedo 66054, Nuevo Leon, Mexico

**Keywords:** *Aedes albopictus*, *Aedes aegypti*, eggs viability

## Abstract

**Simple Summary:**

Diseases transmitted by *Aedes* mosquitoes, such as dengue, Zika, and chikungunya, are a public health problem of growing global concern. There are several strategies for mosquito control. One of them is the Sterile Insect Technique, which is a method of breeding millions of mosquitoes, where radiation is used in males to sterilize them. Then, the males are released into the wild. These sterile males mate with wild females without having offspring, thus decreasing field populations. However, one of the problems is being able to have a large number of viable eggs for these field operations. Therefore, this study evaluates the temporal viability of Aedes mosquito eggs employing two substances, such as hydrolyzed collagen and Hyalurosmooth^®^. These two moisturizing substances maintained viable *Aedes*. *aegypti* eggs for up to eight weeks.

**Abstract:**

Dengue and other *Aedes*-borne diseases have dramatically increased over the last decades. The Sterile Insect Technique (SIT) has been successfully used as part of integrated pest strategies to control populations of insect-plant and livestock pests and is currently being tested as a potential method to reduce mosquito populations in an environmentally friendly approach. However, during the mass rearing steps needed to produce millions of mosquitoes, egg storage and preservation are essential for a certain amount of time. Eggs of *Aedes* *aegypti* have a chorionic pad that functions as a sticky substance to glue them onto the inner walls of larval breeding sites. The chorionic pad is chemically made of hyaluronic acid, a hygroscopic compound, responsible to protect them from desiccation over time. Two commercial products with hygroscopic properties, hydrolyzed collagen, and Hyalurosmooth^®^, both were tested to assess their ability to prolong egg life storage for *A*. *aegypti* and *A*. *albopictus*. Results showed that 85–95% of *Ae*. *aegypti* eggs were able to hatch up to week 8 after being treated with both hydrophilic compounds, compared with the control 66.3%. These two substances showed promising effects for keeping *Ae. aegypti* eggs viable during prolonged storage in mass rearing insect production focused on vector control SIT programs.

## 1. Introduction

The diseases caused by arboviruses have increased over the last decades. Anthropogenic impacts on the environment have allowed a more widespread distribution of many arthropod vector species and the creation of niches with humans [1,2]. Mosquito populations tend to increase due to several factors, including the misuse of chemical products and the high growth of human populations among other things [3]. Species such as *Ae. aegypti* and *Aedes albopictus* benefit from artificial larval habitats of urban areas and propagate disease while feeding on human blood. Hence, they are of great importance for public health [4,5].

The Partnership for Dengue Control (PDC), an independent initiative with multiple sponsors, has been supporting a growing consensus among the dengue prevention community that no single intervention will be sufficient to control the disease. Even if an effective vaccine is available, complementary control strategies of *Aedes* will continue to be necessary [6,7].

The inordinate use of chemical insecticides can become potentially harmful not only for humans but for the environment as well. In addition, the vectors have developed resistance [8]. New mosquito population control strategies are needed and environment-friendly biocontrol strategies, such as the sterile insect technique (SIT) and the Incompatible Insect Technique (IIT), currently receive much attention and are included in the toolkit against Aedes populations. SIT is a method that involves irradiation, for example with gamma rays and X-rays, to sterilize male insects. These males are released in the field to mate with wild females causing egg embryo death. While IIT is based on the sterility that results from the cytoplasmic incompatibility between uninfected wild females and released males infected with a Wolbachia strain that induces this reproductive disorder. Both techniques involve mass rearing management, in which it is necessary to maintain a high egg production rate for colony maintenance [9].

Both SIT and IIT face several challenges, such as finding new sexing techniques and making mass rearing more efficient and cost-effective for the release of millions of mosquitoes. The storage and preservation of *Aedes* eggs for long periods is important for both R&D and SIT field applications [10]. The eggs of *Aedes* mosquitoes have a chorionic pad that functions as an adhesive on the walls of the larval habitats where they are deposited and protect them from desiccation over time because it is made of hyaluronic acid, a hygroscopic compound [11]. We hypothesized that preserving *Ae*. *aegypti* and *Ae*. *albopictus* eggs using chemical substances with the same water-binding capacity would facilitate enhanced moisture retention in the eggs, extending their viability while used in the mosquito rearing process. Therefore, this study aims to assess the ability of two hygroscopic substances to maintain viable and high-quality *Ae*. *aegypti* and *Ae*. *albopictus* eggs as long as possible over 8 weeks.

## 2. Materials and Methods

### 2.1. Mosquito Rearing

The mosquito colonies of *Ae*. *aegypti* and *Ae*. *albopictus* belonging to genetically diverse laboratory strains used previously [12] were separately handled in cages of 28.5 × 28.5 × 28.5 cm (BioQuip^®^ Rancho Dominguez, CA, USA) under controlled conditions of temperature (26 ± 1 °C), relative humidity (RH) (75 ± 5%) and a photoperiod of 14 h:10 h light-dark with a ratio of females and males of 3:1. Both species were provided with a sugar solution of 10% ad libitum and after 5 days post-emergence, females were artificially fed with bovine blood using a Hemotek^®^ membrane feeding system (PS6B, Hemotek Ltd., Great Harwood, UK). Once oviposition took place, the eggs were retrieved and then reared in plastic trays of 29 × 43 × 8 cm with a capacity of 4500 larvae in 3 L of water (density = 1.5 larvae per mL) with conditions of a temperature of 27 ± 1 °C, a photoperiod of 14 h:10 h light-dark and fed with a liquid laboratory rodent diet at 4% (LabDiet, Fort Worth, TX, USA).

### 2.2. Treatments

The substances used for this research were hydrolyzed collagen (Droguería Cosmopolita, CDMX, Mexico, Lot number 899164) and Hyalurosmooth^®^ LS 8998 (Droguería Cosmopolita, CDMX, Mexico, Lot number 0019452763), both with water-binding capacity and hygroscopic properties applied in the fabrication of cosmetics, such as skin humectants and hair treatments. The hydrolyzed collagen composition consisted of a mixture of peptides rich in glycine, proline, and hydroxyproline (https://www.cosmotienda.com/tienda/grenetina-hidrolizada-kgr-p-4024.html) (accessed on 10 February 2021), while the Hyalurosmooth^®^ LS 8998 is made of water, *Cassia angustifolia* seed polysaccharide (galactomannans), and Hydroxyethylcellulose (https://www.cosmotienda.com/tienda/activo-basf-hyalurosmooth-p-4520.html) (accessed on 10 February 2021). For treatments, all solutions of hydrolyzed collagen were water-based at 1%, 3%, and 5%, while Hyalurosmooth^®^ solutions had concentrations on dry weight at 0.42%, 0.83%, and 1.67% from a stock solution of 2.5%.

### 2.3. Egg Collections

After 48 h of the first feeding, a couple of cups were introduced for oviposition in each cage which contained 100 mL of dechlorinated water and a filter paper strip. Forty-eight hours later, recipients were taken out of the cages and the obtained strips were placed for embryonic development over a wet bed for 48 h. Once this time was elapsed, the strips were dried over brown paper for three days.

Once the strips were dried, the eggs were taken gently off using a brush and were then deposited on a Petri dish with white paper in the bottom. The first screening was carried out to remove damaged eggs and impurities, such as mosquito debris, fibers, or other particles. In total, fourteen batches with approximately 4000 eggs each were formed, seven for *Ae*. *aegypti* and another seven for *Ae*. *albopictus*. Each egg batch was distributed in the following treatments for each species: hydrolyzed collagen at 1%, 3%, and 5%; Hyalurosmooth^®^ at 0.42%, 0.83%, and 1.67% and control.

The application of each treatment was performed after inspecting the eggs under a stereoscope, removing damaged and collapsed eggs, and keeping them healthy and viable (round eggs without deformations). The batches were sprayed with atomizers (Miniso^®^ 3.7 cm × 12.9 cm, the volume capacity of 65 mL) using their respective treatments following these steps: (1) a first spray at a distance of 45 cm, (2) two sprays at 30 cm, (3) dried for 45 min, and (4) stored in vessels of 20 mL without light at 27 ± 1 °C and a relative humidity of 75 ± 5%. The control batches were not sprayed or treated and were also stored under the same treatment conditions.

The egg preservation was assessed at long periods (1–8 weeks) of time. After the time had elapsed, the eggs were hatched as follows: (1) three samples of 100 eggs were randomly chosen for each treatment, (2) each sample of eggs was introduced in a tube with 50 mL of a solution of mouse liquid diet (LabDiet 5001) at 0.04% previously incubated for 24 h at 27 ± 1 °C and an RH of 75 ± 5%, (3) then the tubes were closed and gently shaken for 1 h, and (4) the eggs were observed under stereoscope checking if larvae hatched successfully and the egg hatch percentage was finally recorded.

### 2.4. Statistical Analysis

General Linear Models (GLM) were constructed for each species using normalized data under Johnson transformation of percent hatch with a *p*-value of selection of 0.1 utilizing Minitab 17^®^ software (Minitab Inc., State College, PA, USA). The factors considered in the models were treatment, concentration, storage times, and repetitions. To predict and compare how long the preservation effect lasts among the treatments and doses, probit analysis was carried out to obtain the media hatch time (HT50) using the statistical package Statgraphics Centurion 16.2.04^®^ (Statgraphics Technologies, Inc., The Plains, VA, USA). Additionally, the goodness-of-fit χ_2_ for each probit model was calculated with the same software. All analyses were carried out at the 0.05 significance level.

## 3. Results

### 3.1. Hatching Tendency of Eggs over Time

**Figure 1:**
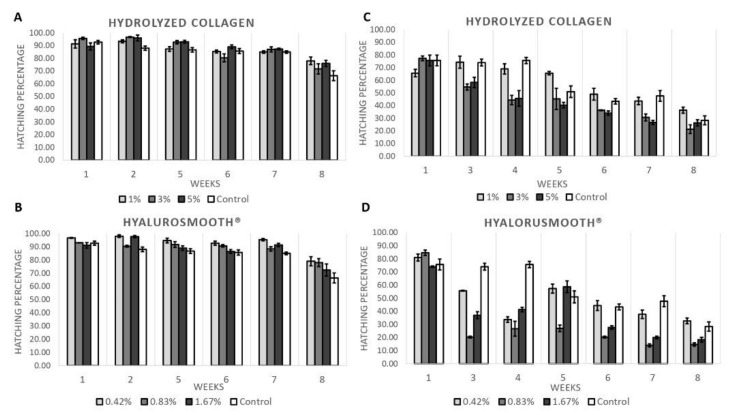
Hatching of *Ae. aegypti* (**A**,**B**) and *Ae. albopictus* (**C**,**D**) eggs preserved during 8 weeks under storage conditions of 27 ± 1 °C and relative humidity 75 ± 5% with exposition to Hyalurosmooth^®^ and hydrolyzed collagen with their respective controls under an SIT program in southern Mexico.

The protective effect of two hygroscopic substances Hyalurosmooth^®^ and hydrolyzed collagen on the preservation potential of *Aedes* eggs during 8 weeks was investigated. Figure 1 shows that the hatching of both *Ae*. *aegypti* and *Ae*. *albopictus* eggs were maintained at higher percentages during the whole period (eight weeks) of this study. Figure 1A exhibits that the eggs had a good resistance of around 85–95% of hatching in *A*. *aegypti* during the first seven weeks, with similar results among treatments and control. However, in the eighth week, the control had a hatching rate of 66.3%, while the Hyalurosmooth^®^ and hydrolyzed collagen treatments showed a higher percentage between 70–80% (Figure 1B). This pattern was not observed in *Ae*. *albopictus* eggs. As shown in Figure 1C,D, the egg hatch rate was reduced compared with the control. *Ae*. *albopictus* treatment with hydrolyzed collagen is only like controls at 1%, with 3% and 5% causing a faster drop off in hatching, like, but not as dramatic as, the toxic effect of Hyalurosmooth^®^. Rates in all three groups are like the control only at 8 weeks. Although the model is not significant, these trends could be noted here. This would strengthen any argument that *Ae*. *albopictus* responds differently to the addition of compounds and must be stored differently to *Ae*. *aegypti*. Similarly, treatments with hydrolyzed collagen presented similar rates as those of the control (Figure 1C). On the other hand, Hyalurosmooth^®^ treatments at 0.83% and 1.67% were even toxic for *Ae*. *albopictus* eggs over time, while at a concentration of 0.42%, the egg hatching rates were similar to those of the control (Figure 1D). It should be noted that during the eight-week experimental period, the percent egg hatching in the control samples was between 65–90% in *A*. *aegypti* (Figure 1A,B) and between 25–75% in *Ae*. *albopictus* (Figure 1C,D).

### 3.2. Dependency of Hyalurosmooth^®^ and Hydrolyzed Collagen on Aedes Eggs

According to the GLM analysis for *Ae*. *aegypti* data (Table 1), a dependency of treatments was observed (F-value: 10.5, *p* < 0.05), and only Hyalurosmooth^®^ treatments showed a statistically significant difference (Coefficient 0.3719, *t*-value: 4.48, *p* < 0.05), while hydrolyzed collagen treatments did not have a significant difference (Coefficient 0.0381, *t*-value: 0.46, *p* = 0.647) when compared with the control. The positive coefficient of Hyalurosmooth^®^ indicates the protective effect of this substance on preserving *Ae*. *aegypti* eggs. For the construction of GLM for *Ae*. *albopictus*, only information up to the sixth week was considered because the percent hatch data of treatments and control of the last two weeks were too low, and if they were included, the data would not have achieved the normal distribution under Johnson transformation. Although *Ae*. *albopictus* had a dependency of treatments (F-value: 15.23, *p* < 0.05), a toxic effect was observed in Hyalurosmooth^®^, presenting a negative coefficient (Coefficient −0.4656, *t*-value: −5.52, *p* < 0.05), while in hydrolyzed collagen, treatments did not have a significant difference (Coefficient 0.0525, *t*-value: 0.59, *p* = 0.556) when compared with the control (Table 1).

### 3.3. Temporal Viability of Aedes Eggs

The degree of effectiveness for each concentration could not be established under the GLM because this factor did not fit in the final models. As *Ae*. *aegypti* was the only species that had a positive effect on egg viability under a GLM, a further probit analysis was carried out to compare and forecast the temporal viability of eggs using different doses of Hyalurosmooth^®^ and hydrolyzed collagen with the control over time. This analysis evidenced that the median hatch time (HT50) of 131.8 days (χ_2_ = 77.8, *p* < 0.05; 95% CI: 111.79–163.16 days) for Hyalurosmooth^®^ at 0.83% was statistically higher than 91.7 days (χ_2_ = 183.7, *p* < 0.05, 95% CI: 82.8–103.4 days) for the control (Table 2). In addition, these probit models were the only ones that showed an adequate goodness-of-fit (Table 2). In the case of hydrolyzed collagen, the HT50 values of 99.1 (95% CI: 88.86–113.02 days) and 99.7 days (95% CI: 87.47–110.81 days) for concentrations at 1% and 3%, respectively, did not show significant differences with the control overlapping the confidence intervals (CI) at 95%. Although the CI for hydrolyzed collagen at 5% did not overlap with the control CI, the model for this concentration did not adequately fit the data (χ2 = 16.1, *p* < 0.05) (Table 2).

## 4. Discussion

A sterile insect technique (SIT) program using irradiated Aedes mosquitoes has been operating in Tapachula, Chiapas, southern Mexico, since 2016 [13]. One of the challenges faced during this program is the reduced viability of *Aedes* eggs when they are stored for long periods waiting for their use in the mosquito-rearing process. The present study evaluated the potential of Hyalurosmooth^®^ and hydrolyzed collagen as treatments to improve the temporal viability of Aedes eggs over eight weeks. Successful results have been achieved with Hyalurosmooth^®^ treatments at 0.42% and 0.83% concentrations but only in *Ae*. *aegypti*, while hydrolyzed collagen did not show a protective effect in *Ae*. *aegypti* nor *Ae*. *albopictus*. Nonetheless, Hyalurosmooth^®^ exhibited a toxic effect in the eggs of *Ae*. *albopictus*.

The protective effect of Hyalurosmooth^®^ may be explained due to its hygroscopic capacity to retain water. It is proposed that the chorionic pad of *Ae*. *aegypti* eggs, made up of hyaluronic acid, have the potential to preserve humidity and avoid desiccation [11,14,15]. The evidence of our results strengthens this hypothesis because of the HT50 of *Ae*. *aegypti* eggs are raised from 91.7 days of control up to 131.8 days using Hyalurosmooth^®^ at 0.83% concentration, an increase of approximately 40 days according to the probit analysis. However, applying this compound at higher concentrations may be counterproductive as seen at 1.67% with a reduced HT50 of 107.3 days. A possible explanation is that at higher concentrations, there could be an alteration in osmotic pressure that could have reduced the hatching instead of promoting it [16,17]. In addition, an excess of polysaccharides of the Hyalurosmooth^®^ may partially interfere in the oxygen exchange in *Aedes* eggs or the egg micropyle [18]. Additional explanations to *Ae*. *albopictus* hatching results could be related to several factors, such as insufficient oxygen levels, hydro- or lipophobic biochemical nature of outer egg layers, exochorion morphology differences, and even lack of experience of insectary rearing operation procedures.

On the other hand, Hyalurosmooth^®^ had a toxic effect on *Ae*. *albopictus* at concentrations of 0.83% and 1.67%, while at 0.42%, no clear difference was observed compared with the control. Additionally, the percent egg hatching in controls was even lower in *Ae*. *albopictus* than in *Ae*. *aegypti*. One explanation of these results may be for the conditions in which experiments were carried out. A previous study showed that the best conditions for *Ae*. *albopictus* egg hatching was at 21 °C with an RH of 95% which resulted in a percent hatch of 83.6% at week 8, while in similar conditions to our research (27 °C and RH 75%), the percent hatch was only 60.5% at week 8 [15]. In our experiments, the hatching at the same week ranged between 28.3% in the control and 36.3% in hydrolyzed collagen at 1%; therefore, there must be another explanation for this substantial reduction in hatching for *Ae*. *albopictus*. One possible cause for this reduced egg hatch rate may be the methodology used in this investigation. The use of artificial sources of larval foods, such as fish or mouse food, has been reported as effective to reduce the oxygen levels in water before the hatching of both *Ae*. *aegypti* and *Ae*. *albopictus* eggs [13,15]. Nevertheless, a previous study indicated that natural resources, such as leaf litter, are better for egg hatching in both Aedes species [19]. Probably, there was a lack of stimuli for *Ae*. *albopictus* eggs because of the use of an artificial diet source (mouse food) that may not have reduced the levels of oxygen in water enough for the hatching of eggs of this species. Another possible explanation could be the absence of unknown natural compounds that are present in the leaf litter and could have better stimulated the egg hatch of *Ae*. *albopictus* in our study.

In the case of hydrolyzed collagen, there were no statistically significant differences between treatments using this protein and the respective controls in the two Aede*s* species. Although collagen has the potential to be rehydrated and retain moisture, this protein likely has lower hygroscopic power than polysaccharides present in Hyalurosmooth^®^. This may be because collagen has a more complex chemical structure than galactomannans and hydroxyethyl cellulose have fewer water molecule bindings with the triple helix [20]. It is necessary to assess the hydrolyzed collagen with other concentrations and verify if this protein works as a preservative of *Aedes* eggs.

There is scarce information available related to the preservation of *Aedes* mosquito eggs, because most of the investigations focus on the control of both immatures and adults [21]. Nonetheless, a methodology called water storage was recently developed to preserve Aedes eggs using eggs submerged in deionized water. This approach resulted in the hatching of 85% of the *Ae*. *aegypti* and *Ae*. *albopictus* eggs after five storage months of both species, while a dramatic decrease was observed if the time was extended up to 24 weeks [22]. The design of our study just contemplated eight weeks and the best preservation treatments for *Ae*. *aegypti* eggs (Hyalurosmooth^®^ at 0.42% and 0.83%) reached a hatch of around 80%. Besides the difference in the study length and the preservation technique, another possible explanation for these differences may be the methodology in the egg hatching, because the use of bacterial broth by Zheng et al. [22] resulted in a 95% egg hatch rate in *Ae*. *aegypti* as compared to 65% of our study during the same eight weeks. One advantage of our procedure is that there is no premature hatching during the storage time as observed by Zheng et al. [22], which means there is no waste of viable eggs. Overall, loss of water is a lethal effect as the egg’s storage time increased [22]. Collapsed eggs are seen and both storage and hatching rate are strongly correlated. A summary of the results of Zheng et al. [22] are included in the guideline documents “Guidelines for Routine Colony Maintenance of Aedes Mosquito Species, version 1.0”, generated by the Joint Food and Agriculture Organization/International Atomic Energy Agency (FAO/IAEA) [23]. Interestingly, most methods rely on oxygen decreasing using physical and chemical strategies to stimulate hatching, including saturated solutions, such as potassium chlorate and potassium sulfate [24,25]. However, in the case of these two compounds, the potential environmental risks could need to fulfill regulations by the local hazard agencies.

Interestingly, the cost-benefit of using the egg stages to field distribution is very promising to low-income endemic countries. For instance, we calculated the cost of 298.91 $ of Hyalurosmooth (0.42%) and 783.44 $ of hydrolyzed collagen (1%) to treat 60 million of *Ae*. *aegypti* eggs. Although pupae are the preferred stages to sterilize during SIT operations, time and distances for their distribution are limited to closer localities where the irradiation source is located. Likewise, the expansion of new sterilized insect programs will be facilitated by using either irradiated eggs or Wolbachia vertical infection.

## 5. Conclusions

In conclusion, our investigation opens a new research approach for the use of hygroscopic substances that retains humidity and, therefore, keep the *Aedes* eggs viable for longer periods. On the other hand, in nature, female Aedes place a sticky substance to glue individual eggs onto the inner surface of the selected breeding site. These substances, mostly hyaluronic and collagen, increase their weight by 600 times or more after water absorption. Because of this property, they are commonly used as dermal fillers in cosmetology. This chorionic pad provides enough humidity to allow the embryo to mature during the first 72 h. In theory, the lack of the sticky compound would favor the immature egg to sink and eventually die [26]. Additionally, frequent field observations of the *Aedes* vector control program report the phenomenon of partial multiple hatching in house backyards larval sites. It possesses a challenge to surveillance activity, considering that these few unhatched egg populations would be later responsible for reinfestation of the same neighborhoods. Currently, poor results to control arboviral *Aedes* diseases urgently demand to include egg mosquito research beside adult and younger stages.

## Figures and Tables

**Table 1:** Summary of the General Linear Models (GLM) for the evaluation of Hyalurosmooth^®^ and hydrolyzed collagen as preservatives of *Ae. aegypti* and *Ae. albopictus* eggs during 8 and 6 weeks, respectively, in southern Mexico.

	*Ae. aegypti* Analysis of Variance for Hatching			*Ae. albopictus*^¥^ Analysis of Variance for Hatching		
	Degrees of Freedom	F-Value	*p*-Value	Degrees of Freedom	F-Value	*p*-Value
Treatments	2	10.56	0.000	2	15.23	0.000
Storage time	5	28.25	0.000	4	23.91	0.000
Repetitions	2	0.10	0.909	2	0.04	0.957
Treatment × storage time	10	1.11	0.369	8	3.96	0.001
Treatment × repetitions	4	0.23	0.918	4	0.18	0.949
Storage time × repetitions	10	0.29	0.980	8	0.30	0.964
Treatment × storage time	20	0.49	0.962	16	0.35	0.989
repetitions						
Total	125			119		
R^2^	76.59%			55.10%		
	Coefficient/SE	T-value	*p*-value	Coefficient/SE	F-Value	*p*-value
Hyalurosmooth^®^	0.3719/0.0830	4.48	0.000	−0.4656/0.0844	−5.52	0.000
Hydrolyzed collagen	0.0381/0.0830	0.46	0.647	0.0625/0.0888	0.59	0.556

^¥^ Only included up to 6 weeks of treatments.

**Table 2:** Probit analysis for different doses of Hyalurosmooth^®^ and hydrolyzed collagen as preservatives of *Ae*. *aegypti* eggs, in southern Mexico.

	Hatching							
	*n*	Slope ± SE	HT50 (Days)	C.I. (95%)	χ^2^	*p*-Value	Goodness-of-Fit χ^2^	*p*-Value
Hyalurosmooth^®^								
0.42%	33	−0.0123 ± 0.0016	151.03	124.80–195.27	59.791	<0.001	10.234	0.017
0.83%	33	−0.0128 ± 0.0014	131.82	111.79–163.16	77.796	<0.001	3.695	0.296
1.67%	33	−0.0166 ± 0.0015	107.28	94.84–124.77	131.854	<0.001	14.475	<0.001
Hydrolyzed collagen								
1%	33	−0.0185 ± 0.0015	99.11	88.86–113.02	159.577	<0.001	5.978	0.113
3%	33	−0.0184 ± 0.0015	97.40	87.47–110.81	167.889	<0.001	17.630	<0.001
5%	33	−0.0142 ± 0.0014	121.12	104.59–145.79	97.071	<0.001	16.148	<0.001
Control	33	−0.0184 ± 0.0014	91.65	82.79–103.41	183.713	<0.001	1.808	0.613

## Data Availability

Not applicable.
